# Utility of Spectral Filtering to Improve the Reliability of Marine Fauna Detections from Drone-Based Monitoring

**DOI:** 10.3390/s23229193

**Published:** 2023-11-15

**Authors:** Andrew P. Colefax, Andrew J. Walsh, Cormac R. Purcell, Paul Butcher

**Affiliations:** 1 Sci-Eye Pty Ltd., Goonellabah, NSW 2480, Australia; 2 School of Computer Science and Engineering, UNSW Sydney, Sydney, NSW 2052, Australia; 3 Trillium Technologies Pty Ltd., 225 Fullarton Road, Eastwood, SA 5063, Australia; 4 New South Wales Department of Primary Industries, Coffs Harbour, NSW 2450, Australia; 5 National Marine Science Centre, Southern Cross University, Coffs Harbour, NSW 2450, Australia

**Keywords:** drone-based monitoring, marine fauna detection, spectral filtering, machine learning analysis, sightability error, shark-bite mitigation

## Abstract

Monitoring marine fauna is essential for mitigating the effects of disturbances in the marine environment, as well as reducing the risk of negative interactions between humans and marine life. Drone-based aerial surveys have become popular for detecting and estimating the abundance of large marine fauna. However, sightability errors, which affect detection reliability, are still apparent. This study tested the utility of spectral filtering for improving the reliability of marine fauna detections from drone-based monitoring. A series of drone-based survey flights were conducted using three identical RGB (red-green-blue channel) cameras with treatments: (i) control (RGB), (ii) spectrally filtered with a narrow ‘green’ bandpass filter (transmission between 525 and 550 nm), and, (iii) spectrally filtered with a polarising filter. Video data from nine flights comprising dolphin groups were analysed using a machine learning approach, whereby ground-truth detections were manually created and compared to AI-generated detections. The results showed that spectral filtering decreased the reliability of detecting submerged fauna compared to standard unfiltered RGB cameras. Although the majority of visible contrast between a submerged marine animal and surrounding seawater (in our study, sites along coastal beaches in eastern Australia) is known to occur between 515–554 nm, isolating the colour input to an RGB sensor does not improve detection reliability due to a decrease in the signal to noise ratio, which affects the reliability of detections.

## 1. Introduction

Due to increasing disturbances, such as effects of climate change, landscape modification, overfishing, and human-wildlife conflict, the importance of monitoring vulnerable marine fauna is intensifying. To this end, aerial survey methods have been a primary means for detecting large marine fauna and estimating their abundance [1,2]. However, while traditional methods of using human spotters to record their observations from a crewed aircraft still occur [3], in many cases digital sampling is increasingly preferred [4]. This is particularly the case with the relatively recent appearance and development of aerial drones, also referred to as ‘UAV’, ‘UAS’, ‘RPAS’ (see Chabot et al. [5]). Drones are now a common tool in ecology [6,7,8]. Furthermore, with the continued advancement of drone technology, as well as associated digital capture technology, it is anticipated that the effective spatial scales that can be efficiently sampled using drone-based methods will expand [9]. Therefore, drones are likely to increasingly replace traditional methods of marine aerial survey for monitoring the population health of large marine fauna [10].

Drone-based aerial surveys in the marine environment are perceived as being a relatively efficient and reliable method for detecting and identifying coastal fauna, and have been used to assess animal behaviour [11], abundance [12,13,14], population health [15,16], as well minimising the potential for human–wildlife conflict, such as human–shark interactions along coastal beaches [17,18]. Despite the utility, sightability errors that affect reliability of the detections and identifications of marine life, can be apparent and similar to that reported from aerial surveys using crewed aircraft [4,8,17]. This can be particularly problematic in marine fauna surveys, where the detection reliability is governed by factors including water clarity, depth, sea state, sun glare, and sea-surface reflection, as well as animal size, behaviour, and its position in the water column [17,19].

Similar to crewed aircraft surveys, the sightability errors associated with detecting marine fauna can be attributed to ‘availability errors’ or ‘perception errors’ [1,4]. Availability errors occur due to an animal being unavailable for detection at the time of the survey pass. In the marine environment, this occurs when an animal is positioned deeper in the water column than the ‘available’ portion of surface water that can be seen from above, in the given conditions of water clarity. Perception biases occur when an animal in the water column should have been detected (i.e., it was positioned in the ‘available’ upper section of the water column), but it was not detected due to an error in human spotting or machine-learning, rather than external factors [1,4,17]. In many cases, sampling effort can be constrained to favourable locations and conditions, and methods can be employed (particularly in post-processing) to estimate the errors and biases to adjust the detection data for inferring abundance [8,19,20]. Minimising the uncertainty in count data has been, and is, a consistent objective across ecology. However, in situations where detections in real-time are required, such as in drone-based surveys aimed at reducing human–shark interactions, the need to investigate and refine methods to improve the reliability of detections are also apparent.

The efficacy of drone-based shark surveys for reducing human–shark interactions currently relies on a drone pilot detecting (and identifying) sharks correctly in real-time from a telemetry screen, and subsequently taking an appropriate course of action based on whether the sighting is a potentially hazardous scenario or not. Despite overwhelming public support for the method [21], a number of scientific research articles are reporting significant error rates in field detections and fauna identifications, which has obvious implications for the efficacy of drone-based shark surveillance for keeping beach-goers safe [17,22,23]. As with a number of other types of drone-based animal surveys, machine learning tools are currently being investigated to minimise human-induced errors in the real-time detection of sharks and other marine life from drones [24,25,26,27,28]. However, although such methods show initial promise, they are still bound to the same sightability constraints that are imposed due to water clarity and the position of the animal in the water column [17,25]. Therefore, despite the utility of machine learning to improve the reliability of detecting and identifying marine animals in real-time (and in post-analysis), potential methods that may improve the contrast of the animal against the background and potentially increase the ‘availability’ of the animal, such as from using alternative sensor technology, may further reduce error in detections [29,30,31].

The potential for alternative sensors, or wavelength selection, to improve the detectability of submerged fauna has not been thoroughly researched. The overwhelming majority of drone-based surveys in the marine environment use RGB sensors, with some applying polarising filters to these cameras to reduce the effect of sun reflection on collected imagery [32,33,34]. Research into applying distortion correction algorithms and augmenting imagery in post-analysis has also demonstrated to have some improvements with regards image clarity, but can involve resource intensive post-analysis [33,35,36]. Similarly, the added cost and complexity throughout the survey and analysis, often preclude the use of alternative sensors. However, various sensors such as infrared, are increasingly becoming more compact and turnkey [10].

Unlike terrestrial environments and above-water applications where thermal imagery has shown clear advantages for improving detection rates and abundance estimates of fauna [26,37], infrared radiation is highly attenuated in water and has very limited utility regarding submerged fauna [38]. However, research into the use of multi-band sensor technologies, such as multispectral and hyperspectral cameras, to improve the detectability of fauna has indicated potential advantages over standard RGB cameras [29,30,31]. Generally, the use of multispectral and hyperspectral cameras in the marine environment have typically used to aid the classification of sessile organisms through analysis of the additional spectral information [39,40]. For detecting mobile marine animals from the air, optimising the use of specific wavelength ranges of light being sampled by the sensor (such as from multi- or hyperspectral cameras), is thought to have the potential to improve the depth that fauna can reliably be detected (availability error), as well as improve the contrast of fauna against the background. This would facilitate obtaining a greater confidence with regards detection reliability (perception bias). However, such sensors are expensive, often not intuitive to use, and offer low spatial resolution when compared to normal cameras.

Previous research has investigated the contrast of various submerged marine fauna against the surrounding seawater [29] and demonstrated that along coastal beaches of eastern Australia, the greatest spectral difference between fauna and seawater was found to occur in the green colour band for submerged coastal marine fauna (515–554 nm). Therefore, this research aimed to test whether low-cost methods of augmenting typical drone cameras to restrict input to these frequencies can maximise the detection reliability of sharks and improve beach safety. Specifically, we tested whether spectral filtering, and spectral filtering with polarisation, could render improved clarity and reliability in fauna detections over standard unfiltered RGB cameras. This would occur if restricting the passband of the input signal improved the signal to noise ratio by essentially cutting out non-useful information.

## 2. Materials and Methods

### 2.1. Equipment

**Figure 1:**
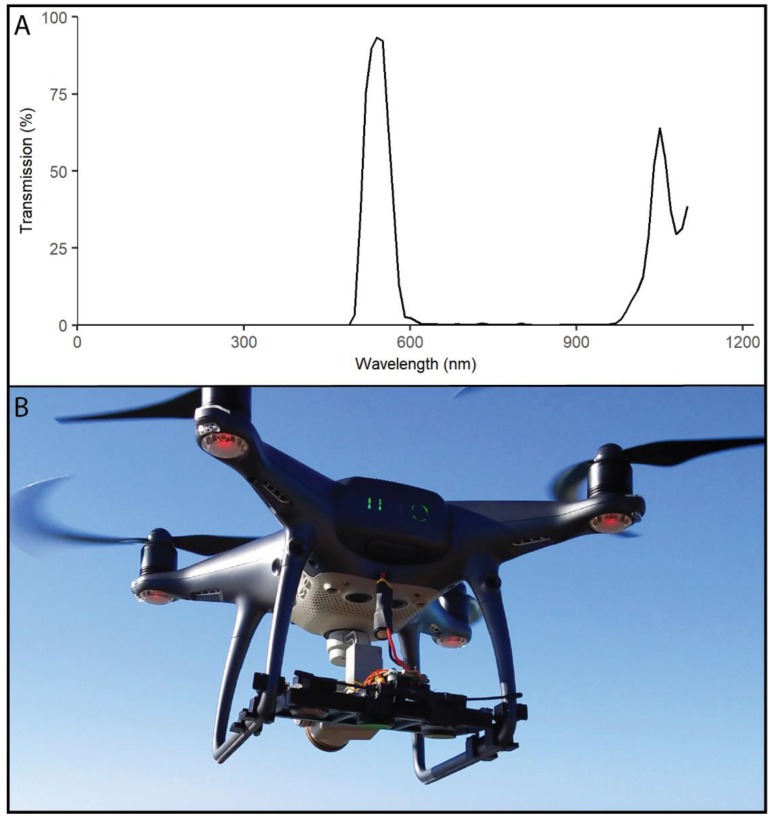
(**A**) The light transmission (%) for the corresponding wavelength (nm) of the narrow green bandpass filters used for filtering treatments. The near-infrared blocking filters (that come standard) in the CMOS sensor of the GoPro negate transmission in the higher (>850 nm) wavelengths. (**B**) GoPro sensor array in the custom mounting, attached to the landing gear of the Phantom 4 Pro drone.

We used a DJI Phantom 4 Pro (1.4 kg drone) due to its portability and versatility in conducting aerial surveys involving multiple flights in remote coastal locations. We attached a small payload to the landing gear of the drone, which enabled comparisons of three camera treatments of identical sensors, including a control, spectrally filtered, and spectrally filtered with polarising filter. Three GoPro Hero8 cameras were used, which we de-cased, minimised, and repackaged with battery-eliminating circuits to be lightweight (~23 g each). They were mounted in a custom housing that attached to flexible vibration dampeners and secured to the landing gear. The GoPro cameras were powered by the drone aircraft battery by a custom auxiliary power plug (Figure 1). A microcontroller with universal asynchronous receiver-transmitter (UART) was used to pair a 915 MHz radio receiver with a transmitter to enable remote start/stop recording of all three GoPros simultaneously. This allowed for a frame-by-frame comparison of video from each of the sensor filter treatments.

Specific narrow-green bandpass filters were fitted to aluminium housings that could be applied to the outside of the two camera lenses. One of the filters had an extra layer of neutral density circular polarising glass. The bandpass filtering glass allowed light transmission specifically between 525 and 550 nm, with a peak transmission of >85% (Figure 1), and minimal angular shifting for the focal length of the GoPro. Angular shifting can occur when objects of interest are away from the centre of frame which can cause wavelength transmission to significantly shift.

### 2.2. Survey

Survey flights were made between July 2021 and August 2022. In these flights, we employed ‘convenient sampling’, and found fauna classes: dolphins (*Tursiops* sp.), sharks (*Carcharhinus* spp.), guitarfish (Rhinobatidae), other rays (*Aetobatus narinari*, *Rhinoptera neglecta*), and whales (*Megaptera novaeangliae*), which have been found along the east Australian coastline in drone-based coastal surveys [18,20,22]. However, dolphins were found to be the most appropriate class for the analysis, due to their vertical movement through the water column.

**Figure 2:**
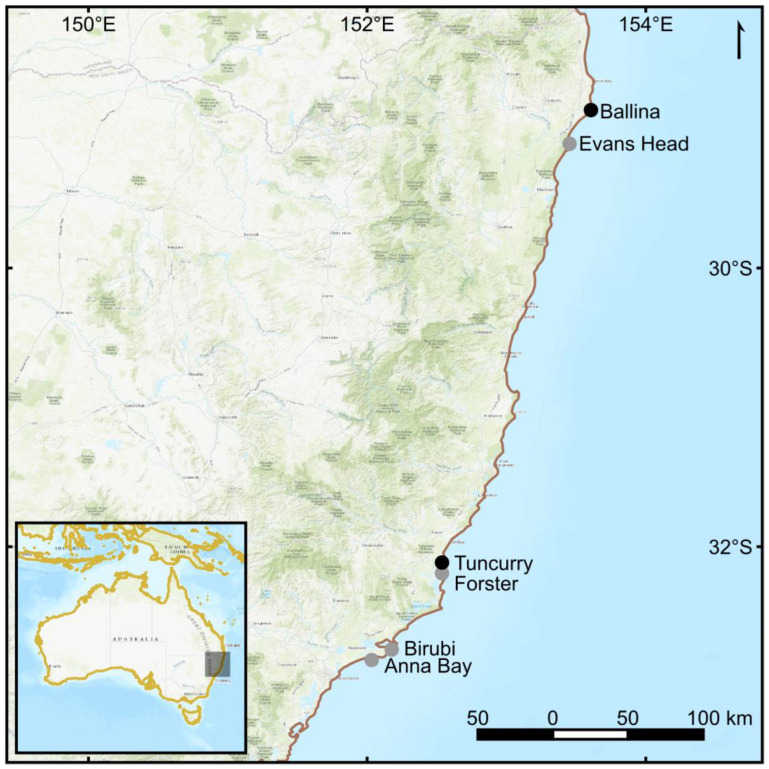
Survey locations on the east coast of Australia. Ballina and Tuncurry represented sites with data that were used in the final analysis. Evans Head, Forster, Birubi and Anna Bay were surveyed but not included in the analysis.

Survey flights were conducted in varied winds of up to around 15 knots (7.7 m s^−^^1^), and in the absence of rain at Ballina, Evans Head, Tuncurry, Forster, Birubi, and Anna Bay on the east coast of Australia (Figure 2). The maximum wind tolerance for sampling was set intentionally lower than the recommended maximum for the aircraft, due to the added payload, which effectively increases the working load on the motors and electronic speed controllers, which require higher amounts of current from the batteries. Care was also taken to fly the aircraft smoothly to avoid unnecessary ‘ramp-up’ of the motors and associated current draw. If wind gusts frequently exceeded ~15 knots, the aircraft was flown back to the ground control station. Flights were made at ~60 m altitude and according to procedures in Colefax et al. [17], using the real-time telemetry of the onboard RGB camera to sight fauna. Flights were made in both directions, following the coastline, just behind the surf break. Once fauna was detected, the drone was lowered to 15–20 m, the camera treatments triggered to record (30 fps at 4k UHD resolution) and the animal/s tracked until it disappeared into the water column, battery was effectively depleted, or weather deteriorated (rainfall or wind >8 m s^−1^). Because the animal was tracked, the trajectory of the drone varied and approximately matched that of the trajectory of the animal. The added payload did impact flight time, with the drone not specifically designed or optimised for the extra weight. Therefore, the maximum wind tolerance for sampling was set intentionally lower than the recommended maximum for the aircraft, due to the added payload, which effectively increases the working load on the motors and electronic speed controllers. This, in turn, draws higher amounts of current from the batteries. Care was also taken to fly the aircraft smoothly to avoid unnecessary ‘ramp-up’ of the motors and associated current draw. If wind gusts frequently exceeded ~15 knots, the aircraft was flown back to the ground control station.

### 2.3. Analysis

A flight was considered successful when we were able to capture marine fauna across all three sensors, in correct exposure, and with sufficient clarity to reliably identify the fauna as it shifted horizontally and vertically in the water column to encompass a range of ‘sightability’ conditions [17,25]. Initial trials of the three-camera setup determined that the cameras recorded within one frame of each other. This was further verified on each video by carefully assessing frame numbers with short-lived events (e.g., the moment a dolphin breaks the surface for air) that were seen by all sensors and could subsequently be used to verify frame-matching across cameras, which enables direct comparisons of the camera treatments.

To empirically contrast the camera treatments of RGB (control), spectral filtering (green filter), and spectral filtering with polarisation, data were analysed using artificial intelligence (AI) deep learning methods. This approach was chosen to eliminate the potential biasing resulting from the subjectivity of using human observation methods to assess the imagery and score for clarity with regards the detectability of submerged fauna. The AI model used as a proxy was an existing RetinaNet single-shot detector (SSD) with a Resnet-50 backbone classifier that was trained on a very large marine fauna dataset (see Purcell et al. [25]). The procedures regarding this previous research for creating the AI model involved carefully annotating (supervised learning) marine faunal datasets that were captured from drone-based shark surveillance trials along coastal beaches of eastern Australia. For each annotation, a set of bounding box coordinates (tightly marking the spatial extent of the animal in the imagery) along with the animal’s classification was recorded with reference to the image. The imagery underwent various augmentation steps to normalise the colour profiles and reduce potential data biases with the animal’s orientation and size through random rotations and image scaling. The training of the model was conducted in a Tensorflow 1.0 environment (now superseded) for ~50 epochs. Training was deemed finished when the mean average precision (mAP), and loss curves (common observational tools to monitor training success of a machine learning model), were observed to flatten out.

**Figure 3:**
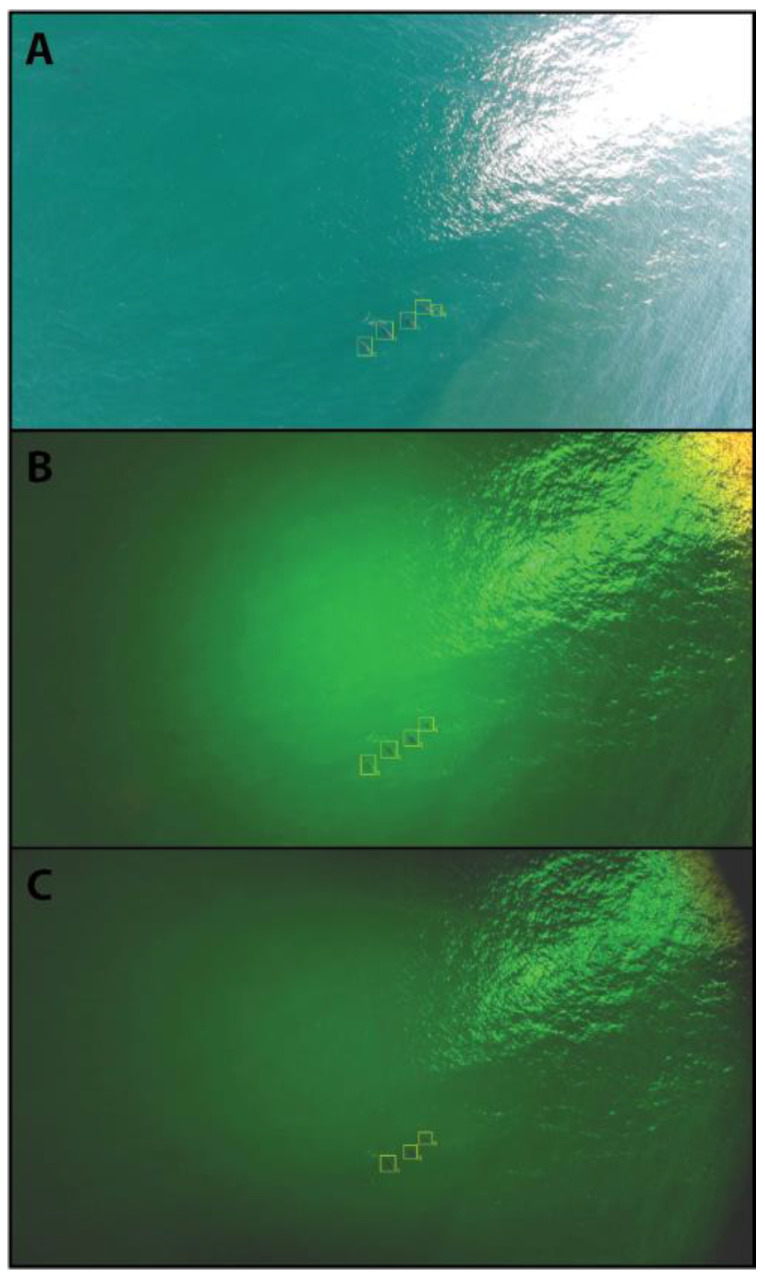
An example of a single frame from each sensor treatment taken on Flight 7, 6 October 2021 at Ballina Headland, NSW, Australia. Top (**A**) shows the unfiltered RGB (red-green-blue channel) sensor, middle (**B**) shows the sensor with green spectral filter, and bottom (**C**) shows the sensor with the green and polarising filter. Each sensor frame is matched in time. Yellow boxes are drawn by the AI model and used for direct comparison of sensor performance.

The approach for the analysis using this previously developed AI model involved first drawing ground-truth boxes over the video footage from each of the camera treatments, and then comparing the ground-truth to boxes generated by the AI model. To create the ground-truth boxes, we manually annotated a select range of frames for each flight using a custom graphical user-interface (GUI) that supported machine learning labelling operations for video. Through the process of ‘boxing’, a coordinate system within each image was generated, which defined the location and bounds of an animal. The box represented the smallest rectangle that can be drawn that completely encapsulated the animal. This was conducted across the range of matching frames for each camera treatment, for each video. The frame range was chosen such that there was no ambiguity in the footage about how many dolphins were present and their locations, which were kept to the middle area of the frame to avoid effects of vignetting or phase shift from the spectral filters (Figure 3). We achieved this by following individual dolphins through the footage from the surface, where they are clearly identified, to being deep in the water column. Where there was more than one dolphin in the frame, each individual was separately tracked and boxed. Animals typically surfaced and dived more than once in a video. When an animal disappeared in the water column (i.e., beyond the sightability threshold), we interpolated boxes for the animal for a number of frames to ensure full extent of sightability was achieved for all three camera treatments.

Once ground-truth boxes were established, we ran inferencing on the videos with the AI model to create AI-generated boxes (Figure 3). Both the ground-truth boxes and the AI-generated boxes were restricted to correspond with cases where animals in the image were not noticeably impacted by phase shifting or vignetting, which was apparent in some scenarios (Figure 3). The AI-generated boxes across the videos were temporally clipped so that the inferencing only corresponded to the same frame sequences of the ground-truth boxes. An important factor for defining whether an AI-generated box matches a ground-truth box is Intersection over Union (IoU), which is a standard measure of the fraction of the AI box that covers the ground-truth box. For a perfect match, the AI and ground-truth box would be exactly aligned, where the IoU would be 100%. However, a partial overlap can also indicate a correct result. We chose a 50% threshold for the analysis based on the results of data inspection (see results). True positive (TP), false positive (FP), and false negative (FN) detections were defined, based on the IoU threshold. The precision (TP/(TP + FP)), and recall (TP/(TP + FN)) were then calculated for each flight and sensor treatment. Precision represents the fraction of all AI-generated boxes that were correct, whereas recall is the fraction of all ground-truth boxes that the AI-generated boxes replicated. These scores were used to contrast the relative performance of each sensor treatment for detecting submerged fauna. The data was analysed and visualised in python.

## 3. Results

We conducted around 86 flights over 18 separate days, totalling 17 h 18 min of total flight time. Survey efficiency, including the locations and timing of surveys, were impacted by weather constraints and COVID-related restrictions, which led to the general scarcity of fauna across surveys. There was a lack of variation in many animals’ vertical position in the water column. This limited the ability to contrast the three camera treatments in many flights. Due to this, we found dolphins to be ideal surrogates for the wider range of fauna classes, as they frequently shift their position in the water column and, in this context, do not spectrally differ from other fauna (see Colefax et al. [29]). Dolphins were also a trained class in the AI model used for the analysis. Due to some technical issues (all three cameras needed to work in sync with the correct exposure), being restricted to specific fauna classes for the analysis, and eliminating cases where the animal in the image may be affected by phase-shifting or vignetting, the dataset was narrowed down to nine successful flights, in a variety of water clarity conditions, on five separate days (Table 1).

**Figure 4:**
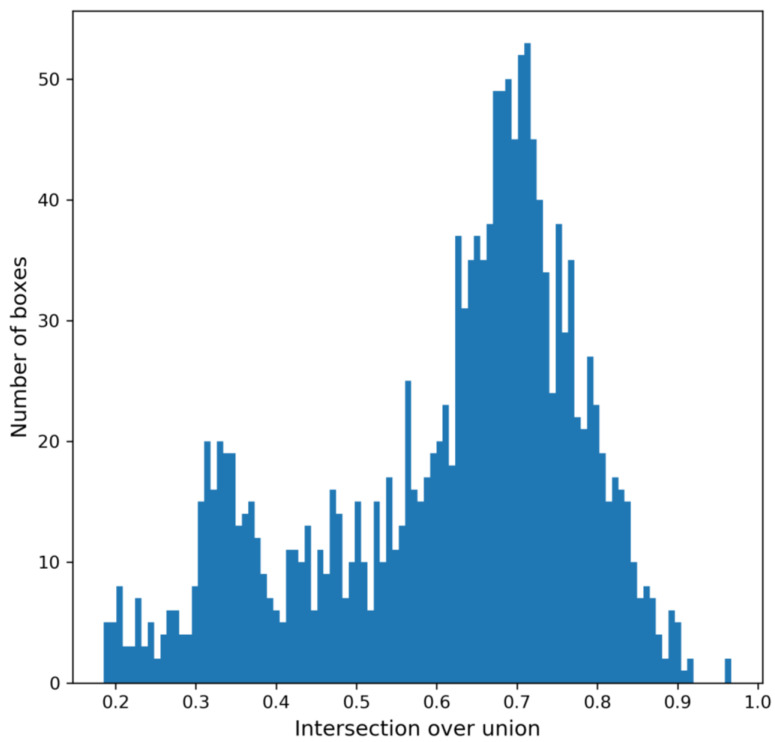
A plot showing an example of a distribution of intersection over union (IoUs) analysis for the RGB (control) sensor for flight 7 on 6 October 2021. The figure shows a clear peak of approximately 70%, with a secondary peak of approximately 35%, which was due to a second dolphin in extremely close proximity. From these plots, the arbitrary IoU of 50% was selected.

The assessments of IoU across frames for each flight showed a general normal distribution that highlighted a 50% threshold would be appropriate to contrast the camera treatments for determining the comparative levels of relative detection performance (Figure 4). While the specific value of IoU can be arbitrary, it is not a definitive measure of detection performance, nor does it bias the overall results when the same IoU threshold is used across all treatments. The AI model was used here as a tool to reliably assess the relative performance of each sensor by setting the threshold for the AI to detect fauna, and then count the number of boxes that the AI model correctly predicts.

**Figure 5:**
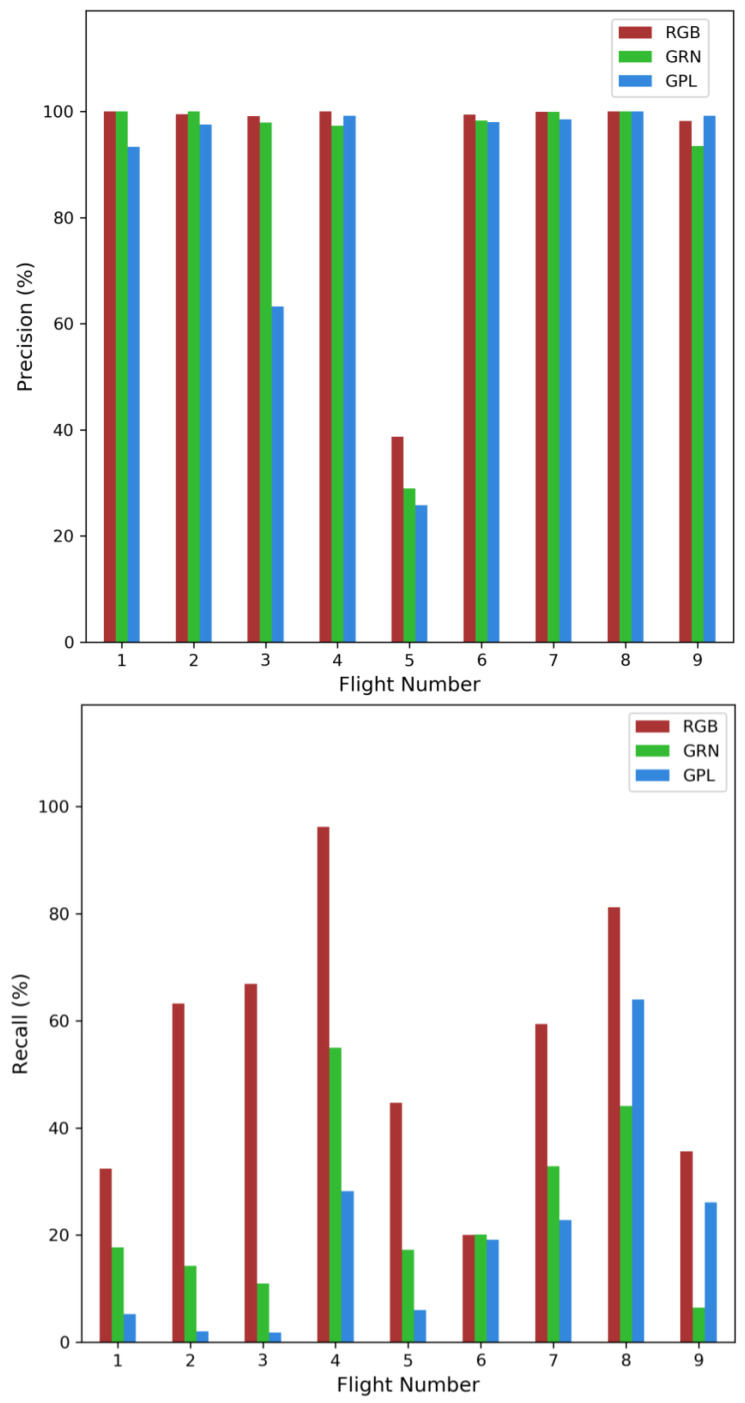
Precision and recall histograms for each flight and each sensor treatment, including RGB (red-green-blue channel), GRN (green filtered sensor treatment) and GPL (green and polarising filter treatment). The highest relative precision and recall (detection reliability) are obtained with unfiltered RGB, while the lowest reliability occur from the GPL sensor treatment.

The distribution of IoUs across flights generally peaked at approximately 74% and followed a normal distribution tailing at ~50% and ~90%. During flights where there was more than one dolphin, a second peak in the distribution was generally found, usually peaking around 35% IoU (Figure 4). This was due to dolphins swimming in close proximity to each other, and subsequently creating overlapping boxes in post-analysis for both ground-truth and AI-generated boxes. Therefore, a true positive (TP) was defined as an AI-generated box that overlapped with a ground-truth box by at least 50%. A false positive (FP) was defined as an AI-generated box that did not overlap with a ground-truth box. A false negative (FN) was defined as a ground-truth box that had no corresponding AI-generated box. The TP, FP, and FN rates were calculated for all flights and camera treatments (Table 2). Because of the nature of the ground-truth boxes, including interpolations that were made for animals beyond the sightability threshold, the precision, recall, and F1 scores relating to the camera treatments are not indicative of AI performance, and reflect relative comparisons between the camera treatments (Figure 5). For more information and an empirical assessment regarding the utility of the AI used in this study for detecting submerged marine fauna along coastal beaches of eastern Australia, see Purcell et al. [25].

The comparisons of precision, recall and F1 score between the sensor treatments indicated that the RGB sensor (average F1 score 66.1 ± 5.0%) consistently outperformed the green sensor (average F1 score 35.8 ± 3.6%), which in turn outperformed the green/polarising filter treatment (Table 2, Figure 5 and average F1 score 28.8 ± 23%).

## 4. Discussion

This study demonstrated that, out of the camera treatments, the best detection reliability results are obtained by using a RGB sensor that is not spectrally restricted, and worst when applying a spectral and polarising filter (in the green band) to an RGB sensor. This is contrary to our initial hypothesis where we expected to see the best performance with the green/polarising filter treatment. The results indicate that the RGB treatment performed better than the green filter treatments. This may suggest that there is useful information captured in the red and blue bands that is lost with applying the green filter. In contrast, while the green/polarising filter was outperformed by the green filter, there is no difference in the wavelength range that the sensors are sensitive to. This may be explained by the fact that only the light of one polarisation is able to pass through to the green/polarised sensor, and the brightness is consequently attenuated by half. Therefore, the signal to noise ratio is also degraded at the sensor, resulting in less information being used to identify a marine animal. This is an unfortunate consequence of light filters, and our results suggest that, whilst narrowing the wavelength range might allow a sensor to ‘focus in’ on the most information-dense part of the spectrum, there is a much larger loss of light from the filter that means the overall performance is degraded.

Detecting submerged marine fauna using drones can be challenging due to various factors such as lighting conditions and the inherent variability of the environment in which the animals reside [1,19,41]. This can cause significant sightability errors which affects the reliability of detections and subsequent identification of target fauna [20,42,43]. To improve this, hyperspectral research investigating the difference in reflectance between fauna and surrounding seawater along coastal beaches of eastern Australia, across 400–1000 nm wavelengths, suggested the vast majority of contrast for detecting fauna was found consistently within the 515–554 nm range [29]. However, the research presented here found that restricting light to these wavelengths entering an RGB sensor worsened the reliability of detections. Furthermore, because the spectral difference of fauna against surrounding seawater in coastal environments does not differ, the results of this study would also apply to other fauna classes. Therefore, if operations are using RGB cameras to spot submerged marine life, then there is no benefit from applying spectral filters. However, other research suggests that polarizing filters can aid in detections by reducing sea-surface reflections [17,42,43].

This study used machine learning to empirically contrast the three camera treatments with arguably less bias than alternative human observation comparison methods. However, as image dimensions are defined as pixels (x, y) as well as layers (one layer for each colour channel), it is evident that the imagery held useful information in the blue and red colour channels. This aided the model in identifying fauna beyond just using the green channel, where the useful information (signal) across all colour channels clearly outweighed the noise [30]. Research on the utility of different spectral bands for detecting submerged whales from satellite showed that, in deeper water, the coastal blue band was superior for detecting southern right whales compared to panchromatic or red-edge bands, and provided the greatest contrast of the animal against the surrounding seawater [30]. However, other studies have performed spectral processing of spatial information from narrow bands (20 nm bandpass), from the blue (~480 nm), as well as green (~535 nm) and red (~600 nm) and reported enhanced detection of submerged whales over standard RGB imagery [31]. This highlights that, although the majority of contrast or ability to detect an animal from surrounding seawater is mostly reliant on a fairly narrow band between the blue and green wavelength range (which would depend on water characteristics of the region of sampling), there is useful information in the broader spectrum (~400–600 nm) beyond the spectral range of the majority of contrast. The greater spectral resolution gained from targeting specific wavelengths of light across the visible spectrum from a multi- or hyper-spectral sensor may offer superior spectral processing and an overall advantage for the detection reliability of submerged fauna than standard RGB sensors for a given spatial resolution. However, research has been proof-of-concept and empirical comparisons between RGB imagery and multispectral cameras are currently scarce [31,44].

It has been recognised that machine learning can reduce the bottleneck that is often encountered during the post-analysis of imagery, and offer further benefits in reducing some sample biases and improving the reliability and consistency of detections [4]. Machine learning can also offer valuable real-time decision support, where detection and classification reliability have direct and immediate implications, such as drone-based surveys for reducing shark-bite mitigation [22,24,25,27,28]. For the analysis of multiband imagery, it is possible that providing spectral processing to the imagery (i.e., enhancing, augmenting or weighting colour channels in a mixing model) can improve the overall detection response [35]. This may also assist as a data pre-processing step for machine learning applications [25]. However, if concerning RGB imagery, standard image augmentation processes already adjust the input data and provide weightings in the colour channels through model training. Although it is likely that adding channels from a multi-hyper-spectral array would provide more information and result in a potentially more reliable model for detecting fauna, the implementation of object-detection methods on sensors that provide high numbers of colour bands can be extremely resource intensive. This presents significant challenges, particularly for on edge or real-time applications [22,25,27]. This would either result in poor inference time, a need for expensive computing hardware, or a compressed model, which could undermine the benefits of having multiple input channels.

The RGB cameras used in this study (GoPro Hero 8 cameras) were not designed for spectral filtering, as applied in the filter treatments. A side effect of this was an effective reduction in the overall light hitting the sensor and an imbalance in the expected colour channel inputs, potentially effecting the colour mixing model applied in the camera. A way to help counteract this was to fix the white balance at 5600 K. However, due to the reduced light, even within the green colour channel, passing through the filter to the sensor, meant that the sensor required the exposure and sensitivity to be increased. This potentially impacted the quality of the image due to exposure compensation of the camera and could bias the results to some degree, particularly with regards to the green polarising filter treatment. Therefore, there may be an improvement in the comparative performance of spectral filtering, such as around 514 and 554 nm for coastal water [29] with sensors more capable of handling wavelength restriction across the colour spectrum (such as panchromatic sensors). However, based on the results of this study, filtering panchromatic lenses (or similar) is unlikely to lead to improvements in detection reliability beyond what can be achieved from standard RGB cameras.

This study demonstrated that, although the majority of visible contrast between a submerged marine animal and surrounding seawater occurs in relatively narrow colour bands, such as between 515–554 nm in beach environments in eastern Australia, isolating the colour input to an RGB sensor does not improve detection reliability. Therefore, the signal to noise outside of this range is still high enough to be of benefit to fauna detection. Due to the rapid absorption and scattering of high frequency light, the utility of multispectral cameras likely is restricted to the visible spectrum, and therefore may not provide significant benefits over RGB cameras, unless many colour bands are leveraged, which has implications regarding the utility and application. Further research into spectral processing on imagery to enhance contrast in the image is required, but current technologies in most circumstances are unlikely to increase the range of sightability into the water column beyond what can be achieved by the current range of RGB cameras.

## Figures and Tables

**Table 1:** Summary of successful flights where dolphins were captured in all three sensors. The number of frames shown in the last column refers to the frames that were used in direct comparison between the sensors.

Flight Number	Date	Location	No. Dolphins	No. Frames
1	31 August 2021	Ballina	2	400
2	31 August 2021	Ballina	2	980
3	31 August 2021	Ballina	2	650
4	1 September 2021	Ballina	1	450
5	6 October 2021	Ballina	1	450
6	6 October 2021	Ballina	1	150
7	6 October 2021	Ballina	6	379
8	4 October 2021	Tuncurry	1	1050
9	8 June 2022	Tuncurry	3	300

**Table 2:** Summary of the relative true positives (TP), false positives (FP), false negatives (FN), along with precision (TP/(TP + FP)), recall (TP/(TP + FN)) and F1 score (TP/(TP + 0.5(FP + FN))), corresponding to each flight number and sensor treatment.

Flight No.	Sensor	TP	FP	FN	Precision (%)	Recall (%)	F1 Score (%)
1	RGB	260	0	542	100	32.4	48.9
1	Green	142	0	660	100	17.7	30.1
1	Green/Pol	42	3	760	93.3	5.2	9.9
2	RGB	1240	6	722	99.5	63.2	77.3
2	Green	279	0	1683	100	14.2	24.9
2	Green/Pol	39	1	1923	97.5	2.0	3.9
3	RGB	871	8	431	99.1	66.9	79.9
3	Green	142	3	1160	97.9	10.9	19.6
3	Green/Pol	24	14	1278	63.2	1.8	3.6
4	RGB	434	0	17	100	96.2	98.1
4	Green	248	7	203	97.3	55.0	70.3
4	Green/Pol	127	1	324	99.2	28.2	43.9
5	RGB	247	392	305	38.7	44.7	41.5
5	Green	95	234	457	28.9	17.2	21.6
5	Green/Pol	33	95	519	25.8	6.0	9.7
6	RGB	468	3	1871	99.4	20.0	33.3
6	Green	469	8	1870	98.3	20.1	33.3
6	Green/Pol	447	9	1892	98.0	19.1	31.2
7	RGB	1228	1	841	99.9	59.4	74.5
7	Green	679	1	1390	99.9	32.8	49.4
7	Green/Pol	472	7	1597	98.5	22.8	37.0
8	RGB	853	0	198	100	81.2	89.6
8	Green	463	0	588	100	44.1	61.1
8	Green/Pol	673	0	378	100	64.0	78.0
9	RGB	320	6	580	98.2	35.6	52.2
9	Green	58	4	842	93.5	6.4	12.0
9	Green/Pol	235	2	665	99.2	26.1	41.3

## Data Availability

Data available on request from the corresponding author with the approval from NSW DPI.
